# Multiple configurations of *EGFR* exon 20 resistance mutations after first- and third-generation *EGFR* TKI treatment affect treatment options in NSCLC

**DOI:** 10.1371/journal.pone.0208097

**Published:** 2018-11-27

**Authors:** Michael E. Goldberg, Meagan Montesion, Lauren Young, James Suh, Joel Greenbowe, Mark Kennedy, Giuseppe Giaccone, Wallace L. Akerley, Afshin Dowlati, Benjamin C. Creelan, James K. Hicks, Paul J. Hesketh, Karen L. Kelly, Jonathan W. Riess, Vincent A. Miller, Philip J. Stephens, Garrett M. Frampton, Siraj Ali, Jeffrey P. Gregg, Lee A. Albacker

**Affiliations:** 1 Foundation Medicine Inc., Cambridge, Massachusetts, United States of America; 2 Department of Oncology, Lombardi Comprehensive Cancer Center, Georgetown University, Washington D.C., United States of America; 3 Huntsman Cancer Institute, Salt Lake City, Utah, United States of America; 4 University Hospitals Seidman Cancer Center, Cleveland, Ohio, United States of America; 5 H. Lee Moffitt Cancer Center & Research Institute, Tampa, Florida, United States of America; 6 Lahey Health Cancer Institute, Lahey Hospital & Medical Center, Burlington, Massachusetss, United States of America; 7 University of California Davis Cancer Center, Sacramento, California, United States of America; 8 Department of Pathology and Laboratory Medicine, UC Davis Health, Sacramento, California, United States of America; University of South Alabama Mitchell Cancer Institute, UNITED STATES

## Abstract

After sequential treatment with first- and third-generation *EGFR* tyrosine kinase inhibitors (TKIs), *EGFR*-mutant non-small cell lung cancers frequently harbor multiple resistance mutations in exon 20 of *EGFR* including T790M, mediating resistance to first-generation TKIs, and at codons 792, 796, or 797 mediating resistance to third-generation TKIs. However, whether these resistance mutations are in *cis* or *trans* has therapeutic implications for patients. We analyzed a cohort of 29 patients with NSCLC harboring *EGFR* mutations at codons 792, 796, or 797 to establish the configuration of these mutations. We performed hybrid capture-based, next-generation sequencing on formalin-fixed paraffin-embedded biopsy tissue or liquid biopsy. 27 samples had both a T790M mutation and a mutation at codons 792, 796, or 797. In all of these cases, the mutations were found in the *cis* configuration; the *trans* configuration was not observed. Two patients’ samples harbored a mutation at codon 797 but no T790M mutation. In these two cases, longitudinal analysis showed earlier biopsies harbored *EGFR* T790M, which was undetectable following osimertinib treatment. Treatment of one these patients with both first- and third-generation *EGFR* TKIs resulted in a mixed response. Here we describe multiple configurations of *EGFR* T790M and third-generation TKI resistance mutations at codons 792, 796, and 797. These mutations are most commonly found in *cis*, which confers resistance to all current *EGFR* TKIs. We also describe two patients that exhibited T790M loss with acquisition of a mutation at codon 797. In addition, one of these patients, with an *EGFR* C797S in a lung biopsy was subsequently found to have *EGFR* C797N in a later biopsy of pleural fluid, highlighting the dynamic multiclonal nature of advanced NSCLC.

## Introduction

*EGFR* tyrosine kinase inhibitors (TKIs) have exhibited clinically significant therapeutic responses in non-small cell lung cancer (NSCLC) patients with tumors that harbor the most frequent *EGFR* driver mutations [1–3]. However, these clinical responses are often short lived, and patients commonly relapse after acquiring an *EGFR* T790M mutation [4]. As a result, a third generation of TKIs towards *EGFR* were developed that preferentially bind mutant *EGFR* proteins in a covalent manner [5, 6]. The most frequent mutations in *EGFR* that cause resistance to third-generation TKIs substitute the cysteine at codon 797 in exon 20, which prevents covalent binding [7]. Additional mutations in exon 20 at codons 792 and 796 have also been shown to confer resistance to third-generation TKIs [8]. Several mutations in other exons of *EGFR* have also been recently identified to confer third generation EGFR-TKI resistance (L718/G719) [9].

After treatment with available EGFR targeted therapies, a tumor could potentially harbor three co-mutations within *EGFR*: a driver, T790M, and third-generation TKI resistance mutation, which has therapeutic and drug development implications. Preclinical work has shown that cells with T790M and C797S mutations on different copies of *EGFR* (in *trans*) remain sensitive to first- and third-generation TKIs in combination, while cells with T790M and C797S mutations on the same copy of *EGFR* (in *cis*) are resistant to all current EGFR TKIs [10]. Two patients have been described with T790M and C797S mutations in *trans* who responded to treatment with first- and third-generation *EGFR* TKIs [11, 12]. To better understand the relationship between *EGFR* driver, T790M, and third-generation TKI resistance mutations in exon 20, we analyzed a cohort of 29 patients with NSCLC harboring *EGFR* driver and a mutation at codons 792, 796, or 797.

## Methods and cohort

### Study consent

This study, including the consent procedure, was reviewed and approved by the Western Institutional Review Board (WIRB, Puyallup WA, clientservices@wirb.com). Written patient consent was obtained at the time of testing. Patients were not consented for release of raw sequencing data.

### Sequence analysis

Clinical samples were tested at a CLIA (Clinical Laboratory Improvement Amendments)-certified, College of American Pathology-accredited, and New York State-accredited laboratory (Foundation Medicine, Cambridge, MA) for comprehensive genomic profiling (CGP) by next-generation sequencing [13]. Tissue samples were sequenced as previously described to a median unique coverage >250X [13]. Liquid biopsy samples were sequenced to >5000X unique coverage [14]. To identify the configuration of T790 and third-generation TKI resistance mutations in exon 20, we analyzed sequencing reads for each sample that spanned both loci. The number of reads that harbored mutations at one or both positions was tabulated and used to infer *cis* or *trans* status.

### Data availability

All consented data are within the paper and its Supporting Information files. WIRB has not authorized and patients were not consented for the release of raw sequencing data, which contains potentially identifiable information.

## Results

### Study cohort

**Fig 1:**
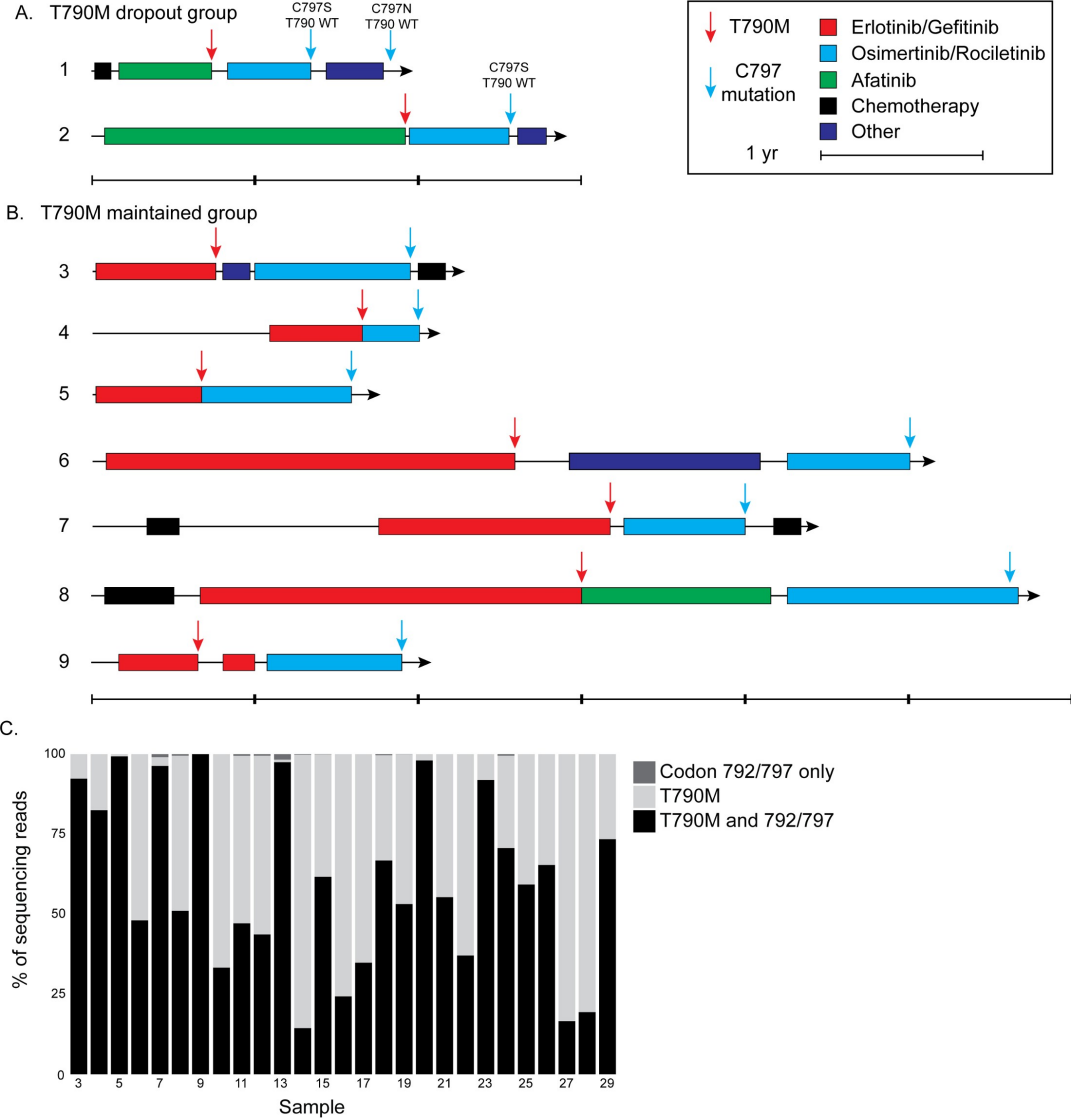
Treatment history and *cis-trans* analysis of NSCLC with co-mutations in *EGFR* at T790M and codon 792 or 797. Time on therapy (filled line) plus emergence of resistance mutations (arrows) is presented for tumors (A) that had T790M loss and (B) those that retained T790M. (C) An analysis of sequencing reads spanning *EGFR* T790 and C797. Only reads with at least one mutation of interest were counted.

We examined a cohort of 29 NSCLC cases with an *EGFR* mutation at codons 792, 796, or 797 (Table 1). The average age of the cohort was 59 and 62% (18/29) were female. Sequencing was performed on FFPE tissue in 22/29 cases and cell free DNA from liquid biopsy in 7/29 cases. We obtained the clinical history of nine patients and confirmed that all nine had received a first or second-generation *EGFR* TKI followed by a third-generation TKI (Fig 1A and 1B and Table 1 and S1 File). Patients responded for 6–28 months on first- or second-generation inhibitors and for 5–16 months on third-generation inhibitors (Fig 1A and 1B).

**Table 1:** Treatment time on *EGFR* TKIs.

Patient	Stage at Dx	1st/2nd gen. TKI	response length (mo.)	3rd gen. TKI	response length (mo.)
1	IV	afatinib	8	osimertinib	5
2	IV	afatinib	23	osimertinib	8
3	IV	erlotinib	9	osimertinib	11
4	IV	erlotinib	6	osimertinib	5
5	IV	erlotinib	8	osimertinib	11
6	IV	erlotinib	19	osimertinib	9
7	IIA	erlotinib	13	rociletinib	11
8	NA	erlotinib	28	osimertinib	16
9	IV	erlotinib	8	osimertinib	10

Dx, diagnosis; gen., generation; TKI, tyrosine kinase inhibitor

We also analyzed longitudinal samples from eight patients. In the cohort with clinical history, CGP of the original biopsy of patients 1 and 5 found the *EGFR* driver mutation (Table 2) but found no mutations at codons 790, 792, 796, and 797. CGP of tissue after relapse on first- and second- generation *EGFR* TKI was performed on patients 1, 6, and 9 and found the original *EGFR* driver mutation (Table 2) in addition to a T790M mutation. In the cohort without clinical history, we performed CGP on longitudinal samples from patients 18, 22, 25, and 28. In these cases, the earliest sample was unmutated at codons 792, 796, and 797.

**Table 2:** Demographics, sample type, and genomic data of patient cohort.

Patient #	External Research #	Gender	Age	Sample Type	*EGFR* drivers	3^rd^-gen resistance
1	TD5S69, QQ2D5K, S7H29M, 8HMKCE	F	53	Tissue	E746_A750del, Amp	C797S, C797N
2	1RVH5H	M	41	Tissue	L747_A750>P, Amp	C797S
3	7M9T6P	F	51	Liquid	E746_A750del	C797S
4	TKAXQ2	M	56	Liquid	E746_A750del, Amp	C797G
5	YVNFS1	M	42	Tissue	E746_A750del, Amp	C797S
6	WRVU0V	M	87	Tissue	L747_A750del	C797S
7	PM529H	F	70	Tissue	E746_A750del	C797S, L792H
8	215P5G	F	60	Tissue	E746_A750del	C797S
9	6ABJSJ	F	75	Tissue	L858R, Amp	C797S
10	8LHYTA	F	63	Liquid	L858R	C797S
11	GUWE2P	F	67	Liquid	E746_A750del, Amp	C797S, L792H
12	1EEUWD	F	74	Liquid	E746_A750del	C797S
13	WDDNPY	F	66	Liquid	E746_A750del	C797S
14	GJDK56	M	53	Liquid	E746_P753>VS	C797S
15	MS3CVN	M	56	Tissue	E746_A750del, Amp	C797S
16	5SVPNA	F	65	Tissue	E746_A750del, Amp	C797S
17	C0RAX4	F	38	Tissue	E746_A752del	C797S
18	EV1SUU	M	61	Tissue	E746_T752>VA, Amp	C797S
19	MB70EK	F	65	Tissue	L858R, Amp	L792H
20	TAT94L	F	67	Tissue	L747_S572del	C797S
21	SRSMSQ	F	40	Tissue	L858R	C797S
22	B9RLRF	F	51	Tissue	L858R	C797S
23	WS2L8P	M	63	Tissue	E746_A750del, Amp	C797S
24	9CT8EF	F	59	Tissue	E746_A750del, Amp	C797S
25	QM4AKG	M	43	Tissue	E746_A750del	C797S
26	RB1ECB	F	55	Tissue	E746_A750del, Amp	C797S
27	CMR9NQ	F	52	Tissue	E746_A750del, Amp	C797S
28	WRPQJH	M	66	Tissue	L747_P753>S, Amp	C797S
29	1ELVJT	M	80	Tissue	E746_A750del	C797G

del, deletion; Amp, gene amplification.

### *EGFR* T790M and third-generation TKI resistance mutations are in *cis*

We analyzed the genomic landscape of 29 patients with an *EGFR* driver mutation and a mutation in *EGFR* codon 792, 796, or 797 (Table 2). The most frequent mutation at these codons was C797S, which was found in 26/29 patients. 3 patients also exhibited multiple resistance mutations with a C797S co-occurring with L792H in two cases and a C797S and C797N observed in separate samples from the same patient (Table 2, Patient 1). Although we did not observe any samples in the present cohort harboring a mutation at codon 796, this mutation has been previously described [8]. The primary driver mutation was an *EGFR* exon 19 deletion in 83% (24/29) of samples while 17% (5/29) had an *EGFR* L858R mutation (Table 2). The T790M resistance mutation co-occurred with mutations at 792 and 797 in the same sample for 27/29 patients and 2/29 patients exhibited an initial sample with T790M and a later sample with a mutation at codon 797 but no T790M mutation (Fig 1A).

We assessed whether *EGFR* T790M and mutations at codons 792 or 797 were in *cis* or in *trans* by analyzing sequencing reads that spanned *EGFR* codons 790 to 797 and contained a mutation in codon 790, 792, or 797. In all 27 cases harboring a T790M and a mutation at codon 792 or 797, nearly all reads with a mutation at codon 792 or 797 also contained a T790M mutation (Fig 1C, 14.5–100% of sequencing reads contained both mutations). Nearly all remaining reads contained only a T790M mutation, which is consistent with the emergence of this mutation earlier in treatment. In 10 samples, we detected reads that contained only a 792 or 797 mutation, but these were at frequencies of <2%. Therefore, we concluded that T790M and codon 792 or 797 mutations for all samples were in *cis*.

### Case studies for *EGFR* C797 mutation, T790M loss tumors

In two samples an *EGFR* C797 mutation without a T790M mutation after treatment with osimertinib was identified. Interrogation of multiple biopsies along the treatment history of these two patients showed that these patients relapsed with *EGFR* T790M alterations after afatinib treatment and then subsequently lost the *EGFR* T790M mutation after treatment with osimertinib (Fig 1A).

Patient 1: A 53-year-old woman was diagnosed with stage IV lung adenocarcinoma (T3N2M1). CGP detected an *EGFR* exon 19 deletion and she was started on afatanib and had a partial response. After progression at 8 months, CGP was performed on a new biopsy and showed an *EGFR* exon 19 deletion and T790M mutation. She started osimertinib and had a partial response but had progression at 5 months. CGP on a new biopsy found an *EGFR* exon 19 deletion and C797S mutation, but the *EGFR* T790M mutation was not detected. She was started on gefitinib and osimertinib and tolerated it well. Follow up at 3 months showed a mixed response with 2 lesions progressing, 2 lesions stable and no new lesions. At 6 months, new metastases were observed. Further CGP testing on a pleural fluid sample at 6 months found the original *EGFR* exon 19 deletion with no EGFR T790M or C797S mutation, but instead a C797N mutation.

Patient 2: A 41-year-old man was diagnosed with stage IV lung adenocarcinoma with metastases to the brain. Genomic testing revealed an *EGFR* exon 19 deletion. The patient was treated with afatinib for 23 months until progression. Liquid biopsy testing found the *EGFR* exon 19 deletion in addition to an *EGFR* T790M mutation and *EGFR* amplification. The patient was started on osimertinib and responded well, but progressed after 8 months. The patient stopped osimertinib treatment and started on a clinical trial with a cMet inhibitor. At that time, CGP found the original *EGFR* exon 19 deletion, an *EGFR* C797S mutation not detected in previous tests, and no evidence of the prior *EGFR* T790M mutation.

## Discussion

*EGFR* T790M is the most common resistance mutation to first-generation *EGFR* TKIs [1–3]. These patients then receive third-generation *EGFR* TKIs and can relapse with a third *EGFR* co-mutation at codon 792, 796, or 797 [5–8]. The configuration of *EGFR* T790M and mutations at codons 792 or 797 was in *cis* in 27/27 patient samples with both mutations, suggesting that the *trans* configuration is rare given this treatment. These findings are consistent with data in the phase 1 AURA study where one of five patients were identified with *trans* T790M, C797S [7, 15]. Two isolated case reports describe *trans* T790M, C797S mutations in two patients who benefited from dual therapy with 1^st^ and 3^rd^ generation *EGFR* TKIs [11, 12]. In addition, T790M loss has been reported in 6 of 12 patients treated with rociletinib [16]. Thress et al. also described osimertinib resistance with either T790M loss and wildtype C797, T790M/C797S double mutant, or T790M positive and wild-type C797 [7]. Here, we report another variation on loss of T790M with the acquisition of a mutation at *EGFR* C797. In our cases, presumably the T790M mutation is not completely lost but only undetectable, suggesting that treatment with first- and third-generation *EGFR* TKIs may be an option for these patients. One of the two patients with T790M loss received this treatment and exhibited a mixed response initially with subsequent progression. We speculate that polyclonality of resistance mechanisms may explain the mixed response. While we have focused on mutations in *EGFR*, resistance alterations in other genes, such as *MET* amplification, could render a tumor non-responsive to any *EGFR* targeted therapy [17]. In support of this speculation, we observed switching of resistance mutations from C797S in a lung biopsy to C797N in a pleural fluid biopsy, which highlights the dynamic clonal evolution occurring in treated tumors. Indeed, a patient with 8 different osimertinib resistance mutations was recently described [8].

Recent clinical trials have led to the approval of osimertinib in a first-line setting for *EGFR* mutant NSCLC [18, 19]. Notably, 0/9 patients in the first-line osimertinib phase 1 trial acquired *EGFR* T790M. Therefore, mutations at *EGFR* codons 792, 796, or 797 would arise first and patients may then receive first-generation *EGFR* TKIs with subsequent emergence of *EGFR* T790M, which may affect the likelihood of observing a *cis* or *trans* configuration. Given the complexities of treatment strategy and modes of resistance, interrogating the relationship between *EGFR* T790M and third-generation *EGFR* TKI resistance mutations may be necessary to guide patient therapy.

## Supporting information

S1 File Case studies for *EGFR* T790M, C797 mutant tumors. Text of case studies for patients 3–9.
(DOCX)
