# Supplementary material for: Multiple configurations of EGFR exon 20 resistance mutations after first- and third-generation EGFR TKI treatment affect treatment options in NSCLC
Source: PLoS One. 2018 Nov 27;13(11):e0208097. doi: 10.1371/journal.pone.0208097 (PMC6258560; doi:10.1371/journal.pone.0208097)
Supplement: S1 File — Text of case studies for patients 3–9. (DOCX) [file pone.0208097.s001.docx]

**Supplemental Text: on-line only**

**Case studies for *EGFR* T790M, C797 mutant tumors**

We obtained clinical histories for a subset of patients to demonstrate that *EGFR* C797 mutations arose after third-generation *EGFR* TKI treatment. Patients 3-9 were treated with a first- or second- generation *EGFR* TKI prior to treatment with a third-generation *EGFR* TKI and harbored *EGFR* T790M and C797X mutations (Figure 1B).

Patient 3: A 52-year-old woman was diagnosed with stage IV lung adenocarcinoma. At diagnosis, molecular testing found an *EGFR* exon 19 deletion. The patient was treated with erlotinib and had a partial response with progression at 9 months. A repeat biopsy of a metastatic lesion found an *EGFR* exon 19 deletion and T790M. The patient was put on ASP8723 Phase 2 clinical trial (third-generation *EGFR* inhibitor), but was discontinued after progression at 2 months. The patient was started on osimertinib and had a partial response with progression after 11 months. Genomic profiling from a plasma-based circulating tumor DNA assay (ctDNA) was performed and found the original *EGFR* exon 19 deletion and T790M with a newly acquired C797S mutation.

Patient 4: A 56-year-old man was diagnosed with stage IV lung adenocarcinoma. Molecular testing identified an *EGFR* exon 19 deletion. The patient started on erlotinib with a partial response but progressed after 6 months. Further molecular testing found an *EGFR* exon 19 deletion and T790M. The patient was treated with osimertinib with partial response but progressed at 5 months. At progression, profiling of ctDNA was performed and the previous *EGFR* exon 19 deletion and T790M mutation were identified in addition to a newly acquired *EGFR* C797S.

Patient 5: A 42-year-old man was diagnosed with stage IV lung adenocarcinoma. Molecular testing identified an *EGFR* exon 19 deletion and the patient was started on erlotinib with a partial response and progression at 8 months. At progression, liquid biopsy found the *EGFR* exon 19 deletion and T790M. The patient was treated with osimertinib with complete response but recurred after 11 months. Tissue-based CGP was performed on a new biopsy and found the *EGFR* exon 19 deletion, T790M mutation, and a newly acquired C797S mutation.

Patient 6: An 87-year-old man was diagnosed with stage IV NSCLC. Molecular testing found an *EGFR* exon 19 deletion and the patient was treated with erlotinib with complete response but recurred at 19 months. On repeat biopsy, molecular testing found an *EGFR* exon 19 deletion T790M. The patient was started on a novel EGFR inhibitor in a Phase I clinical trial and had partial response but had progression at 16 months. The patient was switched to osimeritinib, with a partial response for 9 months but then progressed. A tissue biopsy and CGP was performed. The results showed an *EGFR* exon 19 deletion, an *EGFR* T790M mutation, and a newly acquired *EGFR* C797S mutation.

Patient 7: A 70-year-old woman was diagnosed with stage IIA (T2a, N1, M0) lung adenocarcinoma and treated with surgical resection and 4 cycles cisplatin/pemetrexed but progressed after 22 months. Molecular testing of the initial biopsy found an *EGFR* exon 19 deletion. The patient was then treated with erlotinib with a partial response but progressed at 13 months. Molecular testing of a repeat biopsy found an *EGFR* exon 19 deletion and T790M. The patient was treated with third-generation EGFR inhibitor, rociletinib, and had a partial response for 11 months. CGP was performed at the time of progression while on rociletinib and showed an *EGFR* exon 19 deletion, *EGFR* T790M mutation, and newly acquired *EGFR* C797S and L792H mutations.

Patient 8: A 60-year-old woman was diagnosed with locally advanced lung adenocarcinoma. She was treated with adjuvant chemotherapy of cisplatin/etoposide followed by external beam radiation and progressed after 6 months. Molecular testing on the initial biopsy found an *EGFR* exon 19 deletion. The patient was treated with erlotinib with progression after 28 months. Liquid biopsy found an *EGFR* T790M mutation and the patient was treated with afatanib-dasatinib with stable disease for 14 months. Analysis of a new tissue biopsy confirmed the *EGFR* T790M mutation. The patient was switched to osimertinib with partial response for 16 months. CGP of a new tissue biopsy found the previous *EGFR* exon 19 deletion and T790M mutation in addition to a newly acquired *EGFR* C797S mutation.

Patient 9: A 73-year-old woman diagnosed with widely metastatic stage IV lung adenocarcinoma. CGP was performed on the malignant pleural fluid and found an *EGFR* L858R mutation. The patient was started on erlotinib and had a near complete response but progressed after 8 months. Testing of a second biopsy of the primary lesion exhibited high expression of cMET by IHC and *EGFR* L858R and T790M mutations by CGP. The patient was placed on a clinical trial of erlotinib and a cMET inhibitor but progressed over the next 2 months. The patient was then treated with osimertinib and had a partial response for 10 months. At progression, CGP of a second biopsy of the index lesion found *EGFR* L858R, T790M and a newly acquired C797S mutation.
